# Peripheral monocyte transcriptional signatures of inflammation and oxidative stress in Parkinson’s disease

**DOI:** 10.3389/fimmu.2025.1571074

**Published:** 2025-07-23

**Authors:** Aaron D. Thome, Jinghong Wang, Farah Atassi, Jason R. Thonhoff, Alireza Faridar, Weihua Zhao, David R. Beers, Eugene C. Lai, Stanley H. Appel

**Affiliations:** Department of Neurology, Houston Methodist Neurological Institute, Houston Methodist Research Institute, Houston Methodist Hospital, Houston, TX, United States

**Keywords:** Parkinson’s disease, monocytes, inflammation, neuroinflammation, oxidative stress, peripheral biomarkers, neurodegeneration, neuroimmunology

## Abstract

Parkinson’s disease (PD) is a progressive neurodegenerative disorder characterized by dopaminergic neuron loss in the substantia nigra, which is accompanied by immune dysfunction and chronic inflammation. Peripheral monocytes, key players in systemic inflammation, cross the blood-brain barrier and alter PD etiology and progression. To define the role of peripheral monocytes, cross-sectional studies of RNA transcripts isolated from PD monocytes were compared with age- and sex-matched control monocytes. After stratification by Hoehn & Yahr (H&Y) stage, inflammatory transcripts IL-6, IL-1β, ARG1, CD163, and CCR2 were upregulated in PD monocytes and increased with disease burden. Furthermore, PPARGC1A (PGC-1α), GPX4, NFE2L2 (NRF2), and SIRT3 decreased with increasing disease burden, while only SIRT1 expression increased, reflecting oxidative stress and mitochondrial dysregulation. Overall, the PD monocyte transcripts correlated with PD disease burden as monitored by H&Y, UPDRS total, UPDRS Part 3, ADL, and disease duration. This study demonstrated that dysregulation of inflammation and oxidative stress pathways contributed to disease progression in PD. Monocytes may serve as biomarkers for tracking clinical symptoms and could be leveraged as targets for therapeutic intervention.

## Introduction

Parkinson’s disease (PD) is one of the most prevalent neurodegenerative disorders, marked by the progressive loss of dopaminergic neurons in the substantia nigra pars compacta (SNpc). This degeneration leads to motor symptoms such as tremors, rigidity, and bradykinesia, as well as non-motor symptoms, including cognitive impairment, autonomic dysfunction, and psychiatric disturbances (1–3). Clinical and preclinical studies of PD indicate that immune system dysfunction actively contributes to disease progression (4–10). The full impact of these dysfunctional immune constituents on the central nervous system (CNS) in PD remains unclear.

The interplay between these immune pathways culminates in neuroinflammation that can drive neurodegeneration. Imaging studies have documented early microglial activation in the SNpc, which is hypothesized to precede significant dopaminergic neuronal loss (11). The early neuroinflammatory response of microglia, marked by the release of cytokines such as interleukin-1 beta (IL-1β), interleukin-6 (IL-6), and tumor necrosis factor (TNF), along with the subsequent generation of reactive oxygen species (ROS), creates a toxic microenvironment (6, 12–18). Additionally, reactive astrocyte activation in response to inflammatory cytokines shifts astrocytes into a neurotoxic A1 phenotype (18, 19).

Peripheral immune dysfunction, characterized by altered cytokine levels and dysregulated immune cell function, plays a significant role in PD pathogenesis. This dysfunction is evident in monocytes, T cells, and neutrophils, which exhibit abnormal activity and contribute to elevated serum pro-inflammatory cytokine levels (6, 20, 21). CD4+ T cell subsets are dysregulated in PD, with decreases in Th2, Th17, and Treg cells, and an increase in the pro-inflammatory Th1 subset (6, 18, 20, 22–28). More importantly, Treg populations are reduced in PD patients, showing decreased expression of IL2RA/CD25 and FOXP3, along with impaired suppressive function compared to controls. The extent of this Treg dysfunction correlates with the Hoehn & Yahr (H&Y) disease stage (25). Furthermore, major histocompatibility complex class II (MHCII) expression, particularly HLA-DR, is elevated on microglia in neurodegenerative brain regions and increased on peripheral monocytes. This upregulation, along with the presentation of α-synuclein-specific peptides via MHCII, contributes to T cell activation and neuroinflammation (8, 25, 29, 30).

Monocytes play a significant role in peripheral and central inflammation and have been implicated in the neurodegeneration process in PD. Circulating monocyte populations exhibit heightened pro-inflammatory responses to inflammatory stimuli *in vitro*, producing higher levels of IL-1β, IL-6, and TNF (25, 31–34). Genetic studies have linked multiple PD-associated risk loci to monocyte activation, including variants in the human leukocyte antigen (HLA) system, LRRK2, and GBA1 (35, 36). Additionally, transcriptomic analyses of peripheral monocytes from PD patients have revealed dysregulated gene expression patterns (31, 37). In preclinical PD models, modulating monocyte activation or blocking their recruitment into the CNS reduces neuroinflammation and confers neuroprotection (38, 39).

As peripheral monocytes serve as a critical link between systemic inflammation and neurodegeneration, investigating their phenotypic and transcriptional changes in PD patients across disease progression is warranted. Understanding the role of monocytes in PD pathogenesis may facilitate the identification of biomarkers and the development of therapeutic strategies aimed at modulating neuroinflammation.

## Methods

### Recruitment of patients with PD and controls

Patients with PD and age-matched healthy controls were recruited to the study by Dr. Eugene C. Lai at the Houston Methodist Neurological Institute Neurodegenerative Disease Clinic. Written informed consent was obtained from all participants in accordance with a protocol approved by the Houston Methodist Institutional Review Board (IRB ID: PRO00026718), with initial approval granted on July 29, 2020. This protocol has been reviewed annually to ensure continued compliance with institutional and federal research regulations. All PD patients were evaluated using the Movement Disorder Society clinical diagnostic criteria for PD. Disease burden was assessed using validated clinical scales, including the Hoehn and Yahr (H&Y) staging scale, the Unified Parkinson’s Disease Rating Scale (UPDRS), and the Activities of Daily Living (ADL) scale. At the time of blood collection, all PD patients were undergoing standard-of-care dopaminergic therapy. No patients were drug-naïve or required to withhold treatment for the purposes of this study. This approach reflects the real-world clinical heterogeneity of a chronically treated PD population. A total of 62 PD patients (average age 73.3 ± 7.76 years) and 16 age-matched controls (71.9 ± 7.49 years) were included in the study. Participant demographics and clinical characteristics are further detailed in **Supplementary Table 1**.

### Pan monocyte cell isolations

Following peripheral blood collection from PD patients and healthy controls, peripheral blood mononuclear cells (PBMCs) were isolated using a Lymphoprep (Stemcell) density gradient, followed by indirect magnetic isolation of pan-monocytes using the Pan Monocyte Isolation Kit (Miltenyi Biotec), which enriches for classical (CD14++CD16−), non-classical (CD14+CD16++), and intermediate (CD14++CD16+) monocytes.

### RNA purification and RT-PCR analysis

Following pan-monocyte isolation, RNA was extracted using TRIzol reagent, followed by purification with the Direct-zol RNA MiniPrep Plus Kit (Zymo Research). RNA concentration and purity were assessed using a NanoDrop spectrophotometer. Quantitative RT-PCR (qRT-PCR) was performed using a one-step SYBR Green-based RT-PCR kit (Bio-Rad) on a Bio-Rad iQ5 Multicolor Real-Time PCR Detection System. All primers used in this study were commercially validated PrimePCR SYBR Green Assays obtained from Bio-Rad. Primer sequences, assay IDs, and links to validation data, including melt curve profiles and amplification efficiency, are provided in **Supplementary Table 4** to facilitate transparency and replication of results. Relative gene expression was calculated using the 2^ΔΔCt method, with normalization to beta-actin (ACTB) as the reference gene. Fold change values were calculated relative to healthy controls and transformed into log2 fold change (log2FC). Log2FC values and standard errors of the mean for all analyzed transcripts are reported in **Supplementary Table 2**.

### Statistics and analysis

Statistical analyses were performed using GraphPad Prism software. Transcript log2FC data were visualized using box plots and analyzed using Welch’s t-test with Holm-Sidak p-value correction for comparisons between two groups. Analysis of multiple groups was conducted using one-way ANOVA with Sidak’s multiple comparisons test. *Data are presented as mean ± SEM, and p-values were reported following New England Journal of Medicine conventions (p < 0.05, p < 0.01, p < 0.001). Correlations were analyzed using simple linear regression modeling, and Spearman’s correlation coefficient was calculated with corresponding p-values for each correlation measure.

## Results

### Differential expression of inflammatory and immunoregulatory transcripts in peripheral blood monocytes isolated from patients with PD

**Figure 1:**
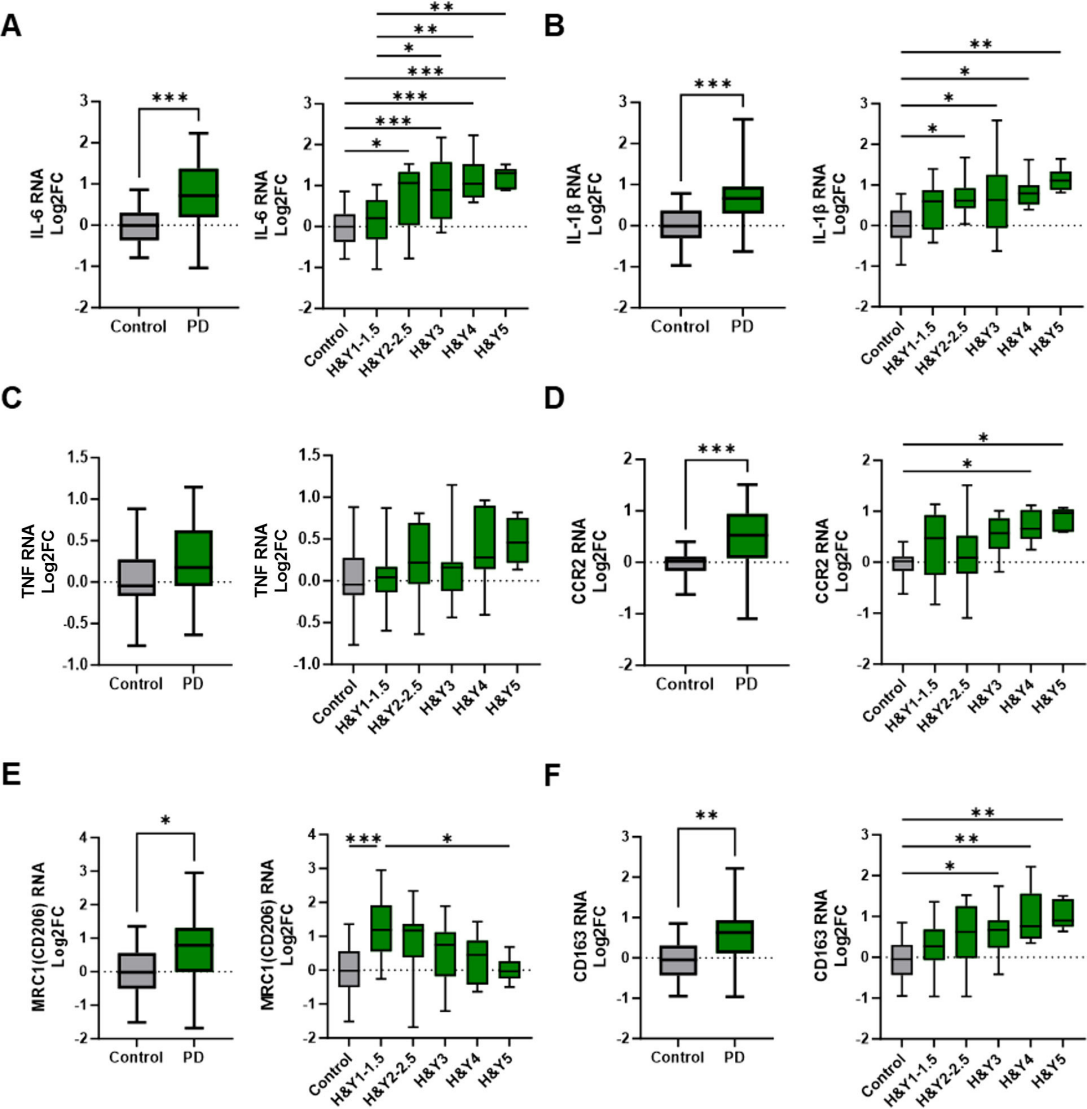
Differential expression of inflammatory and immunoregulatory transcripts in peripheral monocytes from PD patients and through disease progression. Peripheral pan monocytes were isolated from blood of PD patients and controls. **(A–C)** Increased IL-6 and IL-1β pro-inflammatory transcripts in PD monocytes and with increased H&Y stage scoring. **(D)** Cell migration and chemokine receptor CCR2 transcripts increased in PD monocytes. **(E, F)** Increased levels of immunoregulatory markers in PD monocytes compared to controls. Decreasing MRC1/CD206 transcripts with increased H&Y scoring while CD163 transcripts increase with advancing H&Y PD staging. Data shown in **Figure 1** represent log2FC transcript values depicted via box plot summary statistics and analyzed using Welch’s t test with Holm-Sidak p-value correction (PD n=60-62; C n= 16). H&Y data analyzed using ordinary one-way ANOVA with Sidak’s multiple comparisons testing (C n=16; H&Y1-1.5 n=14-17; H&Y2-2.5 n=12-13; H&Y3 n=12-15; H&Y4 n=6-9; H&Y5 n=5-6). Statistical significance read as *p<0.05, **p<0.01, and ***p<0.001).

RNA transcripts involved in inflammation and immunoregulatory pathways were analyzed in peripheral blood monocytes from patients with PD and age-matched controls. These transcripts were then analyzed relative to H&Y stages of disease. Upregulation of the pro-inflammatory cytokine IL-6 transcript was observed in PD monocytes compared to control monocytes (**Figure 1A**), and its expression increased with advancing H&Y stages of PD. Early-stage disease (H&Y 1–1.5) exhibited a modest upregulation of IL-6 compared to controls, while IL-6 RNA levels progressively increased in H&Y 2–2.5, H&Y 3, H&Y 4, and H&Y 5. The pro-inflammatory cytokine IL-1β transcript was also elevated in monocytes from patients with PD and increased with advancing disease (**Figure 1B**), while TNF transcripts displayed a moderate increase but did not change significantly with disease progression (**Figure 1C**). The chemokine receptor C-C chemokine receptor type 2 (CCR2), which facilitates monocyte migration to sites of inflammation, was upregulated in PD monocytes compared to controls (**Figure 1D**). Additionally, CCR2 expression was increased in early PD and continued to rise with advancing disease stages.

Transcripts associated with anti-inflammatory and immunoregulatory functions were also examined in monocytes isolated from patients with PD and healthy controls. Transcripts of the mannose receptor (MRC1/CD206), a marker of alternatively activated (M2) myeloid cells, were upregulated in early-stage PD monocytes but declined with disease progression, resulting in decreased expression in late-stage disease (H&Y 5) (**Figure 1E**). CD163, a scavenger receptor associated with immunoregulation and protection from oxidative stress, was increased in monocytes from PD patients. CD163 transcripts were low in early-stage PD but increased with disease progression (**Figure 1F**). Arginase 1 transcripts (ARG1) were elevated in PD monocytes relative to controls, but their expression varied across disease stages (data not shown).

### Alterations in metabolic, oxidative stress, and mitochondrial regulatory transcripts in peripheral monocytes of PD patients

**Figure 2:**
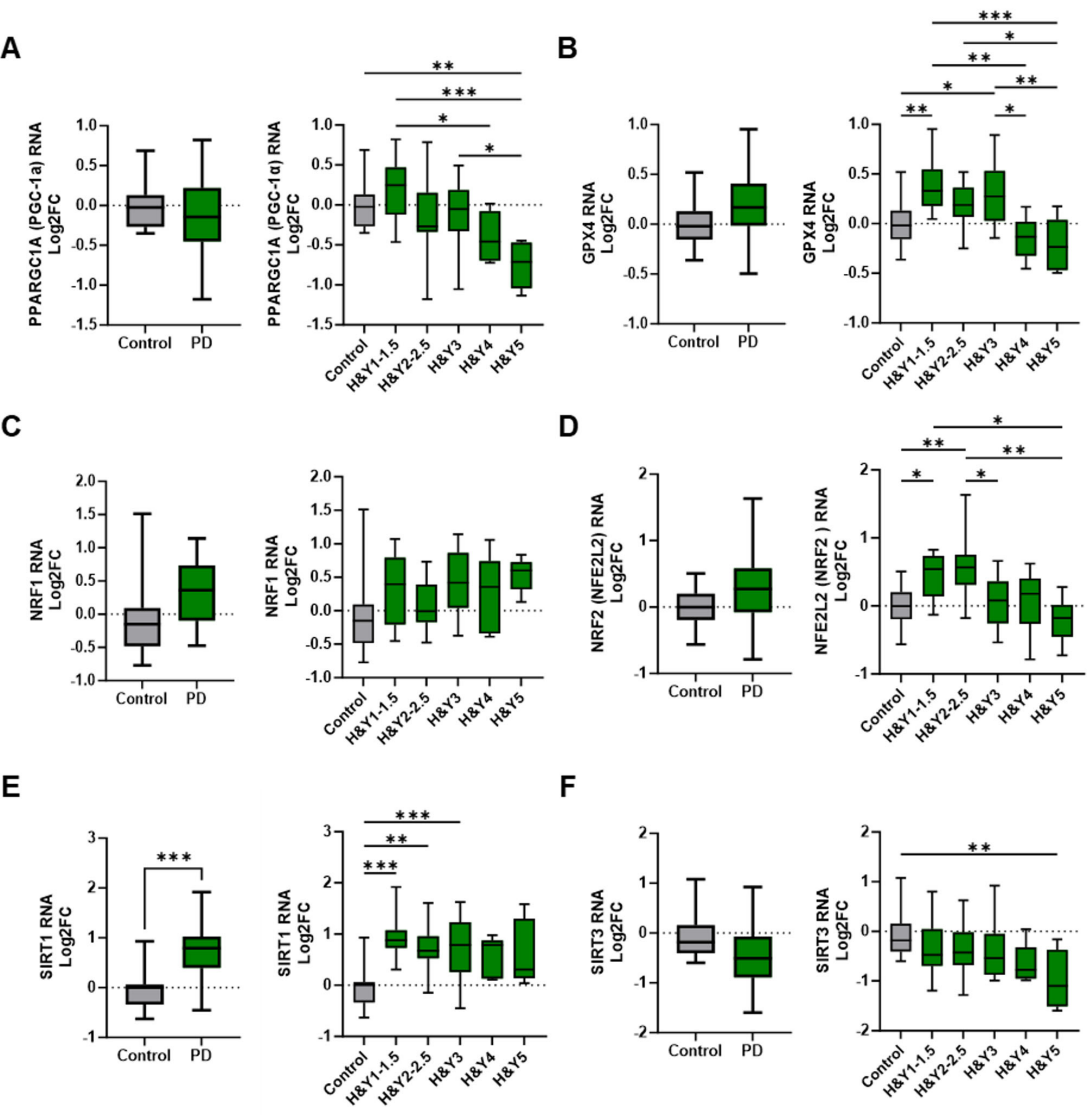
Alterations in metabolic, oxidative stress, and mitochondrial regulatory gene expression in peripheral monocytes of Parkinson’s disease patients. **(A)** Changes in PD monocyte PPARGC1A/PGC-1α gene transcripts compared with controls and through H&Y disease progression. **(B)** GPX4 transcripts in PD vs control monocytes that increased early in PD progression staging. **(C, D)** NRF1 and NRF2 transcripts in PD monocytes and through disease progression staging. Antioxidant NRF2 increased early in PD staging but significantly decreased in later stages. **(E)** Increased SIRT1 expression in PD monocytes with elevations early in PD progression and remaining elevated through intermediate disease. **(F)** SIRT3 RNA is slightly decreased early in PD with a significant drop in late stage of disease. Data shown in **Figure 2** represent log2FC transcript values depicted via box plot summary statistics and analyzed using Welch’s t test with Holm-Sidak p-value correction for PD vs Control analysis (PD n=60-62; C n= 16). Ordinary one-way ANOVA with Sidak’s multiple comparisons testing utilized for H&Y staging of disease analysis (C n=16; H&Y1-1.5 n=14-17; H&Y2-2.5 n=12-13; H&Y3 n=12-15; H&Y4 n=6-9; H&Y5 n=5-6). Statistical significance read as *p<0.05, **p<0.01, and ***p<0.001).

PD is directly associated with aberrant oxidative stress mechanisms, mitochondrial dysfunction, and metabolic dysregulation. Peroxisome proliferator-activated receptor gamma coactivator 1-alpha (PPARGC1A or PGC-1α), an important transcriptional activator, exhibited a slight but non-significant increase in early-stage PD monocytes. However, its transcript levels progressively declined as the disease advanced, with the most pronounced reductions occurring in H&Y 4 and H&Y 5 stages (**Figure 2A**). Glutathione peroxidase 4 (GPX4), an antioxidant enzyme implicated in PD, was elevated in PD monocytes compared to controls (**Figure 2B**). When stratified by H&Y stage, GPX4 expression increased early but declined in later stages of disease.

Nuclear respiratory factor 1 (NRF1) and nuclear factor erythroid 2–related factor 2 (NFE2L2/NRF2) regulate oxidative stress and mitochondrial function, with impairments linked to mitochondrial dysfunction, oxidative damage, and inflammation. While NRF1 transcripts were increased in early-stage PD, overall NRF1 expression did not significantly differ in PD monocytes compared to controls (**Figure 2C**). NRF2 transcripts initially increased at H&Y 1 and H&Y 2–2.5 but declined in stages H&Y 3 through 5 (**Figure 2D**).

Sirtuin 1 (SIRT1) and Sirtuin 3 (SIRT3) are NAD^+^-dependent deacetylases with critical roles in oxidative stress responses, mitochondrial regulation, and inflammation. SIRT1 transcripts were upregulated in PD monocytes relative to controls; expression increased during early and intermediate disease stages but declined with disease progression (**Figure 2E**). Conversely, SIRT3 transcripts were reduced in PD monocytes, with early stage decreases that became more pronounced as disease advanced (**Figure 2F**).

### Correlations between monocyte gene expression and clinical parameters in PD patients

**Figure 3:**
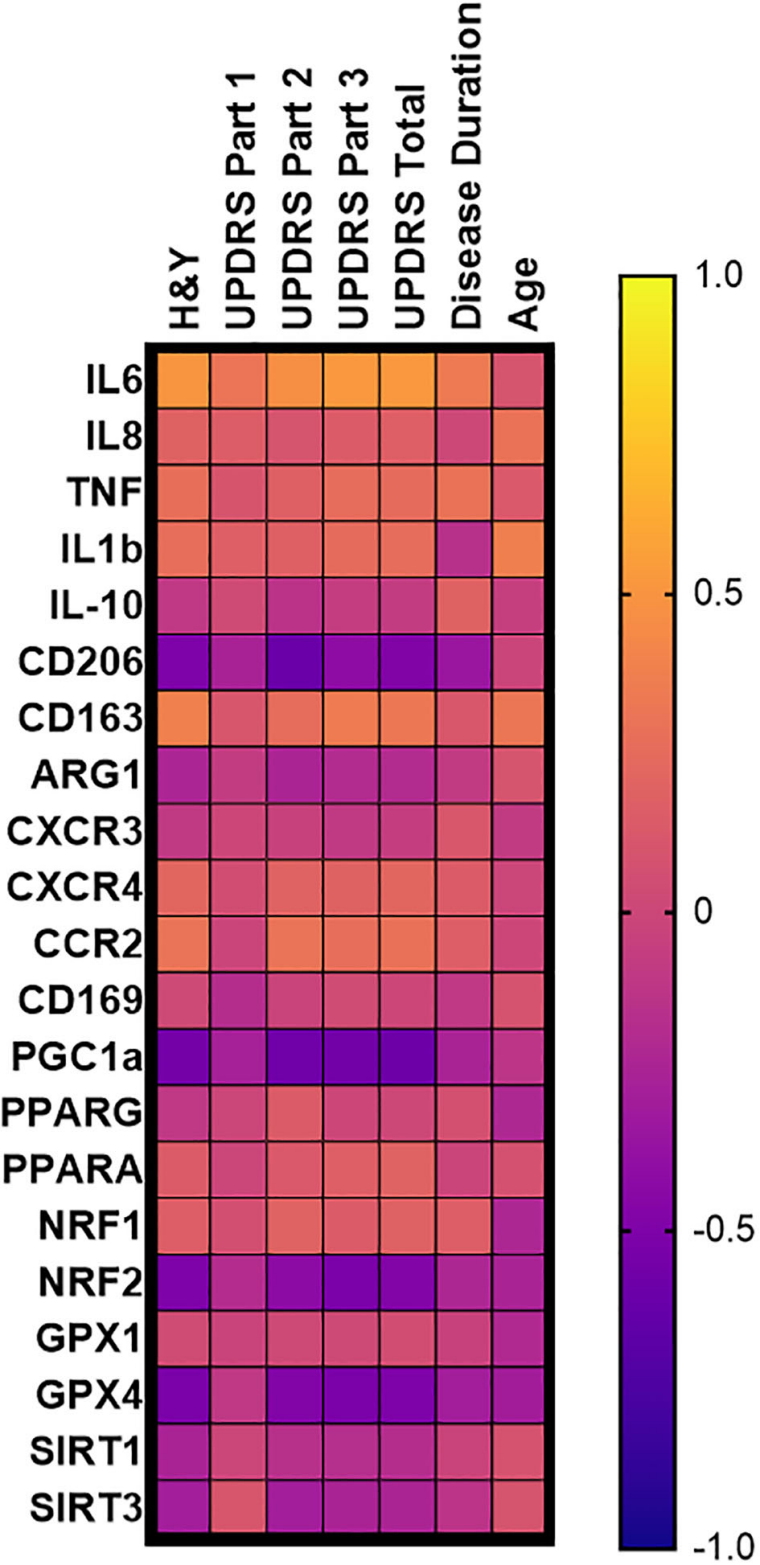
PD monocyte transcript correlation matrix. Correlation matrix of PD monocyte inflammatory and oxidative stress transcripts as a function of common disease burden assessments done in PD patients. Spearman r correlation values calculated using PD monocyte log2FC expression against patient testing parameters of H&Y, UPDRS, disease duration, and age of the patient. Exact correlation values and significance shown in **Table 1**.

Spearman correlations of monocyte transcripts from patients with PD were assessed using multiple clinical measures of disease burden, including H&Y scoring, the Unified Parkinson’s Disease Rating Scale (UPDRS), the Parkinson’s Disease Activities of Daily Living Scale (ADL), as well as measures of disease duration and age (**Figure 3**, **Table 1**). IL-6 transcripts correlated with H&Y progression (ρ= 0.5007, *p*<0.001), UPDRS Part 3 scoring (ρ= 0.5193, *p*<0.001), UPDRS total scoring (ρ= 0.5145, p<0.001), and the ADL scoring (ρ= -0.4324, *p*=0.003) (**Table 1**, **Supplementary Figure 1A**). With respect to anti-inflammatory and phagocytic transcripts, there were negative correlations for MRC1/CD206 transcripts including H&Y scale (ρ= -0.4989, *p*<0.001), UPDRS part 3 (ρ= -0.4119, *p*=0.004), UPDRS total scoring (ρ = -0.4844, *p*<0.001), and disease duration (ρ= -0.3458, *p*=0.02) (**Table 1**, **Figure 1B**). Conversely, CD163 transcripts had positive correlations with H&Y (ρ= 0.3750, *p*=0.009), UPDRS part 3 (ρ= 0.3475, *p*=0.02), and UPDRS total scoring (ρ =0.3213, *p*=0.03) (**Table 1**, **Supplementary Figure 1C**).

**Table 1:** Monocyte Log2FC and disease progression correlation matrix table.

PD Monocytes	H&Y		UPDRS Part 3		UPDRS Total		ADL Score		Disease Duration		Age	
	Spearman r		Spearman r		Spearman r		Spearman r		Spearman r		Spearman r	
Log2FC Expression	ρ	*p* value	ρ	*p* value	ρ	*p* value	ρ	*p* value	ρ	*p* value	ρ	*p* value
IL-6	0.5007	<0.001	0.5193	<0.001	0.5145	<0.001	-0.4324	0.003	0.3334	0.03	0.0842	0.57
IL-8	0.1769	0.22	0.1413	0.34	0.1660	0.26	-0.2151	0.14	0.0113	0.94	0.2926	0.04
TNF	0.2695	0.06	0.2475	0.09	0.2432	0.1	-0.2322	0.11	0.2872	0.05	0.1226	0.4
IL-1β	0.2618	0.07	0.2451	0.1	0.2507	0.09	-0.2753	0.06	-0.1561	0.31	0.3682	0.01
IL-10	-0.0999	0.49	-0.0700	0.64	-0.0815	0.59	0.1003	0.5	0.1841	0.22	-0.0559	0.7
CD206	-0.4989	<0.001	-0.4119	0.004	-0.4844	<0.001	0.5500	<0.001	-0.3458	0.02	-0.0195	0.9
CD163	0.3750	0.009	0.3475	0.02	0.3213	0.03	-0.4179	0.004	0.1113	0.47	0.3135	0.03
ARG1	-0.2388	0.1	-0.1982	0.19	-0.1992	0.18	0.1675	0.27	-0.0838	0.58	0.0845	0.57
CXCR3	-0.0955	0.51	-0.0932	0.53	-0.0603	0.69	0.0388	0.79	0.1101	0.46	-0.0861	0.55
CXCR4	0.2107	0.14	0.1838	0.22	0.2136	0.15	-0.1090	0.46	0.1405	0.35	-0.0061	0.97
CCR2	0.2993	0.04	0.2609	0.08	0.2846	0.06	-0.2026	0.18	0.1577	0.3	0.0036	0.98
CD169	0.0132	0.93	0.0304	0.84	-0.0021	0.99	-0.0729	0.62	-0.1004	0.5	0.0776	0.59
PGC-1α	-0.5542	<0.001	-0.5623	<0.001	-0.5866	<0.001	0.6374	<0.001	-0.2495	0.09	-0.1249	0.39
PPARG	-0.0983	0.5	-0.0008	>0.99	0.0068	0.96	0.0616	0.68	0.0593	0.69	-0.2203	0.12
PPARA	0.1438	0.32	0.1632	0.27	0.1854	0.21	-0.1259	0.39	-0.0193	0.9	0.0701	0.63
NRF1	-0.2518	0.28	0.1391	0.35	0.1762	0.24	-0.0767	0.6	0.1554	0.3	-0.2276	0.11
NRF2	-0.2922	<0.001	-0.5154	<0.001	-0.4791	<0.001	0.4438	0.002	-0.2269	0.13	-0.2528	0.08
GPX1	0.1544	0.83	0.0190	0.9	0.0455	0.76	0.0566	0.7	-0.0405	0.79	-0.2105	0.14
GPX4	-0.5035	<0.001	-0.5190	<0.001	-0.5034	<0.001	0.6135	<0.001	-0.2863	0.05	-0.2989	0.03
SIRTl	0.0311	0.08	-0.1754	0.24	-0.1828	0.22	0.2100	0.15	-0.0273	0.86	0.0777	0.59
SIRT3	-0.5184	0.04	-0.2506	0.09	-0.2465	0.09	0.2393	0.1	-0.1317	0.38	0.0856	0.55

Spearman’s rank correlations (r value) and associated p-values for correlated monocyte log2FC expression values and disease progression parameters such as H&Y, UPDRS total and part 3, ADL score, disease duration, and age. Statistically significant correlations of p < 0.05 are highlighted in red. Positive and negative correlations are represented by sign in front of the r value. Significant correlations suggest potential relationships between the log2FC and the clinical measures in the dataset.

The oxidative stress PPARGC1A/PGC-1α transcripts were negatively correlated for H&Y (ρ= -0.5542, *p*<0.001), UPDRS part 3 and total (ρ = -0.5623, *p*<0.001; ρ= -0.5866, *p*<0.001), and ADL scoring (ρ= 0.6374, *p*<0.001) (**Table 1**, **Supplementary Figure 2A**). There was no correlation for disease duration (ρ= -0.2495; *p*=0.09). As for NFE2L2/NRF2 antioxidant transcripts, similar negative correlations were found with H&Y (ρ= -0.2922, *p*<0.001), UPDRS part 3 (ρ= -0.5154, *p*<0.001), UPDRS total score (ρ= -0.4791, *p*<0.001), and ADL score (ρ= 0.4438, *p*=0.002) (**Table 1**, **Supplementary Figure 2B**). A similar observation was found for GPX4 transcripts with H&Y (ρ = -0.5035, *p*<0.001), UPDRS part 3 (ρ = -0.5190, *p*<0.001), UPDRS total score (ρ= -0.5034, *p*<0.001), and ADL score (ρ = 0.6135, *p*<0.001) (**Table 1**, **Supplementary Figure 2C**). PPARGC1A/PGC-1α, NFE2L2/NRF2, and GPX4 did not correlate with disease duration (**Table 1**).

Most transcripts did not correlate with patient age. Specifically, IL-6 and MRC1/CD206 had correlations with disease duration, but they did not correlate with patients’ age. PPARGC1A/PGC-1α and NFE2L2/NRF2 had correlated with disease progression, but there was no correlation with age. The monocyte transcripts that did significantly correlate with age were CD163 (ρ = 0.3135, *p*=0.03) and GPX4 (ρ= -0.2989, *p*=0.03) while the cytokine transcript IL-1β (ρ= 0.3682, *p*=0.01) did correlate with patient age; however, it did not correlate with disease progression (**Table 1**). Stratifying the Log2FC and disease progression correlations into male and female outputs, it was revealed that females retained a stronger correlative inflammatory signature while both males and females retained strong oxidative stress and mitochondrial dysfunction monocyte correlations (**Supplementary Table 3**).

## Discussion

Immune dysfunction and chronic inflammation contribute to PD pathogenesis and progression, yet identifying peripheral, non-invasive indicators of disease onset and progression remains a challenge. In this cross-sectional study, differential expression of inflammatory, immunoregulatory, and chemotactic receptor transcripts was observed in peripheral blood monocytes isolated from patients with PD, and age- and sex-matched controls. Specifically, there was an upregulation of IL-1β, IL-6, IL-10, MRC1/CD206, CD163, and CCR2 transcripts in PD monocytes. IL-1β, IL-6, CD163, and CCR2 transcripts increased with advancing disease while MRC1/CD206 transcripts were elevated early in disease but declined as disease advanced. Metabolic, oxidative stress, and mitochondrial transcript analysis showed an upregulation of SIRT1 in PD monocytes. Dysregulation of PPARGC1A/PGC-1α, GPX4, and NFE2L2/NRF2 transcripts was evident in disease burden analyses and was increased in early disease PD monocytes but declined in later stages; SIRT1 transcripts were elevated early and throughout disease staging, while SIRT3 declined later in disease. Finally, correlation analyses of monocyte transcripts across multiple PD progression parameters revealed significant correlations with respect to H&Y, UPDRS Part 3, UPDRS Total, and ADL staging and disease duration, but no correlation with the patients’ age.

There is now increased interest in peripheral blood monocytes as both a potential biomarker and a target for therapeutic intervention in PD. Gene expression studies in PD patients have demonstrated that PD-associated risk genes are highly expressed in monocytes, leading to downstream immune-altering effects. Previous studies have reported altered numbers and shifts in peripheral blood monocyte populations, along with upregulated surface expression of HLA-DR. These findings are consistent with a shift toward more pro-inflammatory monocyte subsets (25, 34). These alterations were also shown to be increased with respect to increasing disease burden. When isolated and stimulated ex vivo, PD monocytes exhibit a hyperactive, pro-inflammatory response, producing higher levels of pro-inflammatory cytokines than monocytes from healthy controls (33). In preclinical PD models, peripheral monocyte infiltration into the CNS is a critical step in α-synuclein-mediated neuroinflammation and subsequent neurodegeneration (38, 39). Their entry is thought to exacerbate inflammation, which is toxic to neurons, and blocking their migration into the CNS has been shown to be neuroprotective in these models.

The observed upregulation of pro-inflammatory cytokine transcripts IL-1β, IL-6, and TNF aligns with previous reports documenting the upregulation of these cytokines in the peripheral blood of patients with PD (20, 21). The progressive rise in IL-1β and IL-6 transcripts with increasing disease burden supports the notion that chronic and exacerbating pro-inflammatory signaling occurs in PD. This may serve as a proxy indicator of the toxic microenvironment in the CNS of these patients.

The upregulation of anti-inflammatory transcripts such as ARG1, MRC1/CD206, and CD163 in isolated PD monocytes suggests a potential compensatory response to disease, particularly in early stages. MRC1/CD206 is predominantly expressed on the surface of activated myeloid cells and plays a critical role in the immune response by both enhancing phagocytosis and modulating immune responses to an alternative anti-inflammatory/resolution state (40). CD163 is increased on myeloid cells near neurodegenerative pathology, including PD, while soluble CD163 correlates with exacerbation of inflammation and worsening disease outcomes in neurodegenerative, inflammatory, and autoimmune diseases. Interestingly, CD163 expression on isolated PD monocytes remains steady in early stages of disease and increases with advanced disease burden. This paradoxical effect of CD206 and CD163 may signal dynamic shifts in monocyte activation states during the course of disease. CD206 is associated with an anti-inflammatory phenotype, and the increasing levels of this transcript may reflect an initial anti-inflammatory response aimed at counteracting early neuroinflammation. CD163, also linked to anti-inflammatory phenotypes, does not increase until late stage of disease, and this late response may indicate compensatory mechanisms mitigating oxidative stress and pro-inflammatory responses (40–42). Our finding of increased CD163 transcripts in PD monocytes with advancing disease may reflect a compensatory upregulation in response to enhanced proteolytic shedding of membrane-bound CD163 into the circulation. This mechanism may serve to replenish surface expression amid ongoing monocyte activation. Prior studies have demonstrated that soluble CD163 (sCD163) levels rise progressively with PD severity and correlate with markers of neuroinflammation and neurodegeneration, further supporting the role of CD163 as a dynamic indicator of monocyte activation and disease progression (43).

Additionally, CCR2 expression on isolated peripheral blood PD monocytes was most pronounced in advanced stages of disease. Increased CCR2 expression is associated with enhanced migratory capacity of monocytes to the CNS. CCR2 ligands are elevated in patients with PD and lead to enhanced migration and infiltration of pro-inflammatory monocytes to the CNS which may contribute to toxic neuroinflammation with subsequent neurodegeneration (44–48). Blocking CCR2 recruitment pathways to the CNS reduces monocyte infiltration to the CNS, abrogates neuroinflammation, and prevents neurodegeneration (39).

PGC-1α is a master regulator of mitochondrial biogenesis and antioxidant defenses, protecting myeloid cells from pro-inflammatory activation and promoting an anti-inflammatory, M2 phenotype (49–51). The current data demonstrated that PGC-1α transcripts were elevated early but declined with increased disease burden. PGC-1α is known to be decreased in CNS tissues from patients with PD and in preclinical models of disease which may drive mitochondrial dysfunction, increasing oxidative stress and pro-inflammation responses. Targeting PGC-1α in preclinical models of PD induces neuroprotective benefits, suggestive of a promising therapeutic intervention for PD (49, 50, 52).

Oxidative stress, mitochondrial dysfunction, and lipid peroxidation are documented driving forces in PD pathogenesis and progression (53–56). Examination of these pathways in myeloid cells is limited, but monocytes have been shown to enter and directly contribute to CNS pathology (46–48). Myeloid cells are early and preferentially vulnerable to oxidative and ferroptotic stress, and may serve as a proxy for the CNS microenvironment while sampled from the blood (57). Of significance is the current demonstration that GPX4 and NRF2 transcripts were increased in early disease but then declined at later stages. GPX4 is an essential enzyme that protects cells from lipid peroxidation, ferroptosis, and subsequent cell death. A functional loss of GPX4 in late-stage PD may exacerbate lipid peroxidation and ferroptotic stress (58, 59). Early increases in NRF2 transcripts may be indicative of an adaptive response to early oxidative stress, but its decline may signal a compromised antioxidant defense system (60).

Sirtuin family transcripts are also altered in isolated PD monocytes. SIRT1 is increased early in PD, but its expression is lost by mid to late-stage disease resulting in loss of its anti-inflammatory effects on NF-kB signaling and autophagy. SIRT3, another sirtuin deacetylase known to mitigate mitochondrial dysfunction and promote antioxidant activity, is modestly decreased early, and is further decreased in the late-stage disease. Transcriptional changes in sirtuins have been reported to contribute to PD pathogenesis through their roles in inflammation regulation, oxidative stress mitigation, and mitochondrial enhancement/maintenance (61–63). SIRT1 and SIRT3 dysregulation have been primarily studied in dopaminergic neurons, but their altered expression in peripheral monocytes suggests their potential as biomarkers and therapeutic targets.

Patient sex differences are a biological variable that affects the immune system, whether that be its function or potential dysfunction in disease-associated patient monocytes (64–66). The current study had a relatively equal male-to-female ratio. When stratifying the correlation parameters based on sex, male monocytes lost some of the strength in the inflammatory correlations (**Supplementary Table 2**). Conversely, females retained and strengthened their correlative Spearman r value with respect to the inflammatory monocyte transcripts. In a different study examining peripheral immune cells in PD, female monocytes demonstrated greater inflammatory activation, while the male monocyte activation patterns were more heterogeneous (67). The predominance of males over females in a PD diagnosis, along with the heterogeneity of myeloid cell activation states in the males, may complicate the tracking of peripheral myeloid inflammatory changes in patients with PD. The observed sex-specific transcriptional correlations likely reflect fundamental immunological dimorphisms, including the influence of sex hormones, differential regulation of immune signaling pathways, and X-linked gene expression. These dimorphisms have been reported in both health and disease, with female immune cells often exhibiting heightened inflammatory responsiveness and more robust transcriptional activity (68, 69). Clinically, such differences may contribute to sex-based variability in PD disease presentation or trajectory, as males are more commonly diagnosed, yet some studies suggest females may experience more rapid cognitive and functional decline (70). These findings underscore the importance of considering sex as a biological variable in PD immune biomarker research. Interestingly, the oxidative stress and mitochondrial transcript signatures remained significant across both sexes, supporting the hypothesis that mitochondrial dysfunction in PD monocytes represents a sex-independent and potentially CNS-relevant marker of disease. Future studies are warranted to further define these sex-specific immune alterations through monocyte subset stratification, transcriptomic and epigenetic profiling, and integration with clinical phenotyping to clarify their mechanistic and therapeutic relevance in PD.

These data generate a hypothesis that pro-inflammatory mechanisms and oxidative stress generate a toxic microenvironment that can inhibit anti-inflammatory mechanisms, cause cellular dysfunction of tolerogenic immune cells such as Tregs, and even drive polarization of anti-inflammatory cells to pro-inflammatory responses. With myeloid cells being the main driver of pro-inflammatory mechanisms in the brain and periphery, targeting these cells and enhancing Treg function would be a promising strategy to overcome immune dysfunction.

The current study has several limitations. First, the number of healthy controls was smaller than the PD cohort, which may reduce statistical power for some comparisons; however, the groups were age- and sex-matched, and statistical methods were used to mitigate the effects of unequal sample sizes and variances. Future longitudinal studies and expanded recruitment through Parkinson’s disease consortia will improve group matching and enable more robust analysis of clinical trajectory and immune changes. Second, the use of a pan-monocyte isolation approach effectively captures total monocyte transcriptional responses but precludes analysis of subset-specific changes in classical, intermediate, and/or non-classical monocytes. Future studies incorporating subset-specific sorting and/or single-cell RNA profiling will be important for refining these findings and correlations. Third, all PD patients were on dopaminergic therapy at the time of sampling. While these therapies are not thought to substantially alter adaptive T cell polarization, their effects on peripheral monocytes are less well understood. Although we did not assess dopamine receptor expression on monocytes, prior studies have shown dopaminergic modulation of T cell receptor profiles during PD progression, and *in vitro* data suggest dopamine can alter monocyte chemotaxis and activation (71, 72). The transcriptomic changes reported here occurred despite ongoing therapy, suggesting persistent monocyte dysregulation. Preclinical models support a role for dopaminergic signaling in promoting myeloid cell trafficking into the CNS and inflammation, and blocking monocyte recruitment in PD models, especially CCR2+ subsets, has shown neuroprotective effects (39, 73–75). These findings support a role for CCR2+ monocytes in PD-related neuroinflammation and highlight the need for future longitudinal studies to track dopaminergic therapy initiation, dosage, and regimen changes to clarify its influence on monocyte phenotypes and their association with disease progression. Lastly, although this cross-sectional study identified correlations between monocyte transcripts and clinical parameters, causality cannot be inferred, and further mechanistic studies are required to determine how these immune changes contribute to PD pathophysiology.

Overall, these data highlight a dysregulation in peripheral monocytes in PD patients, evidenced by both pro-inflammatory transcript activation and dysregulation of oxidative stress and mitochondrial dysfunction transcripts. This dysregulation is particularly striking when looking at the changes across different stages of disease burden; there is an early anti-inflammatory and antioxidant response in the monocytes followed by a more pro-inflammatory phenotype in late-stage disease. These data suggest the importance of myeloid cells in the pathogenesis of PD and the value of circulating cells to monitor inflammation and oxidative stress pathways. Peripheral monocytes may serve as viable biomarkers of both disease and progression, supporting the development of therapeutic agents that target and inhibit their pro-inflammatory activity.

## Data Availability

The raw data supporting the conclusions of this article will be made available by the authors, without undue reservation.

## Supplementary material

Supplementary Figure 1 PD monocyte inflammatory transcripts correlate with different PD progression parameters. To evaluate correlations with different PD progression parameters, we plotted data and applied simple linear regression modeling to visual trend. Spearman’s correlation coefficient (r) with corresponding p value depicted in each graph with respect to H&Y progression staging, UPDRS scoring, and disease duration via years. Examination of previously significant inflammatory or immunoregulatory transcripts of **(A)** IL-6, **(B)** MRC1/CD206, and **(C)** CD163.

Supplementary Figure 2 PD monocyte oxidative stress transcripts correlate with PD progression parameters. Evaluation of correlations with different PD progression parameters were plotted with applied simple linear regression modeling done to visual trend data. Spearman’s correlation coefficient (r) with corresponding p value depicted in each graph with respect to H&Y progression staging, UPDRS scoring, and disease duration via years. Examination of previously significant inflammatory or immunoregulatory transcripts of **(A)** PGC-1a, **(B)** NRF2, and **(C)** GPX4.

Supplementary Table 1 Demographics and Clinical Characteristics of PD Patients Demographic summary table of age, sex, and disease progression of PD patients recruited to the study. Demographics detailed as PD cohort as a whole and separated into male vs female. Disease progression stratified by Hoehn and Yahr (H&Y) scale. Age represented as mean ± standard deviation (SD) and disease duration reported as average years.

Supplementary Table 2 Log2FC values for PD monocytes and H&Y Log2FC values. Summary table of Log2FC averages and standard error of the mean (SEM) of monocyte gene expression levels across different stages of PD via H&Y scale.

Supplementary Table 3 Patient sex stratified correlation table. Sex stratification of the correlation data. Spearman’s rank correlations (r value) and associated p-values for correlated monocyte log2FC expression values and disease progression parameters such as H&Y, UPDRS total and part 3, ADL score, disease duration, and age. Statistically significant correlations of p < 0.05 are highlighted in red. Positive and negative correlations are represented by sign in front of the r value. Significant correlations suggest potential relationships between the log2FC and the clinical measures in the dataset.

Supplementary Table 4 Primer information for transcript analysis. Table describes commercially validated PrimePCR SYBR Green Assays (Bio-Rad) used for quantitative RT-PCR analysis of monocyte RNA transcripts. For each target gene, the table provides the gene symbol, full gene name, Bio-Rad unique assay ID, and link to online validation (sequences, melt curves, performance metrics, etc.). These primers were used to quantify gene expression changes across patient and control samples in this study.
