# Supplementary figures and images for: Peripheral monocyte transcriptional signatures of inflammation and oxidative stress in Parkinson’s disease
Source: Front Immunol. 2025 Jul 23;16:1571074. doi: 10.3389/fimmu.2025.1571074 (PMC12325044; doi:10.3389/fimmu.2025.1571074)

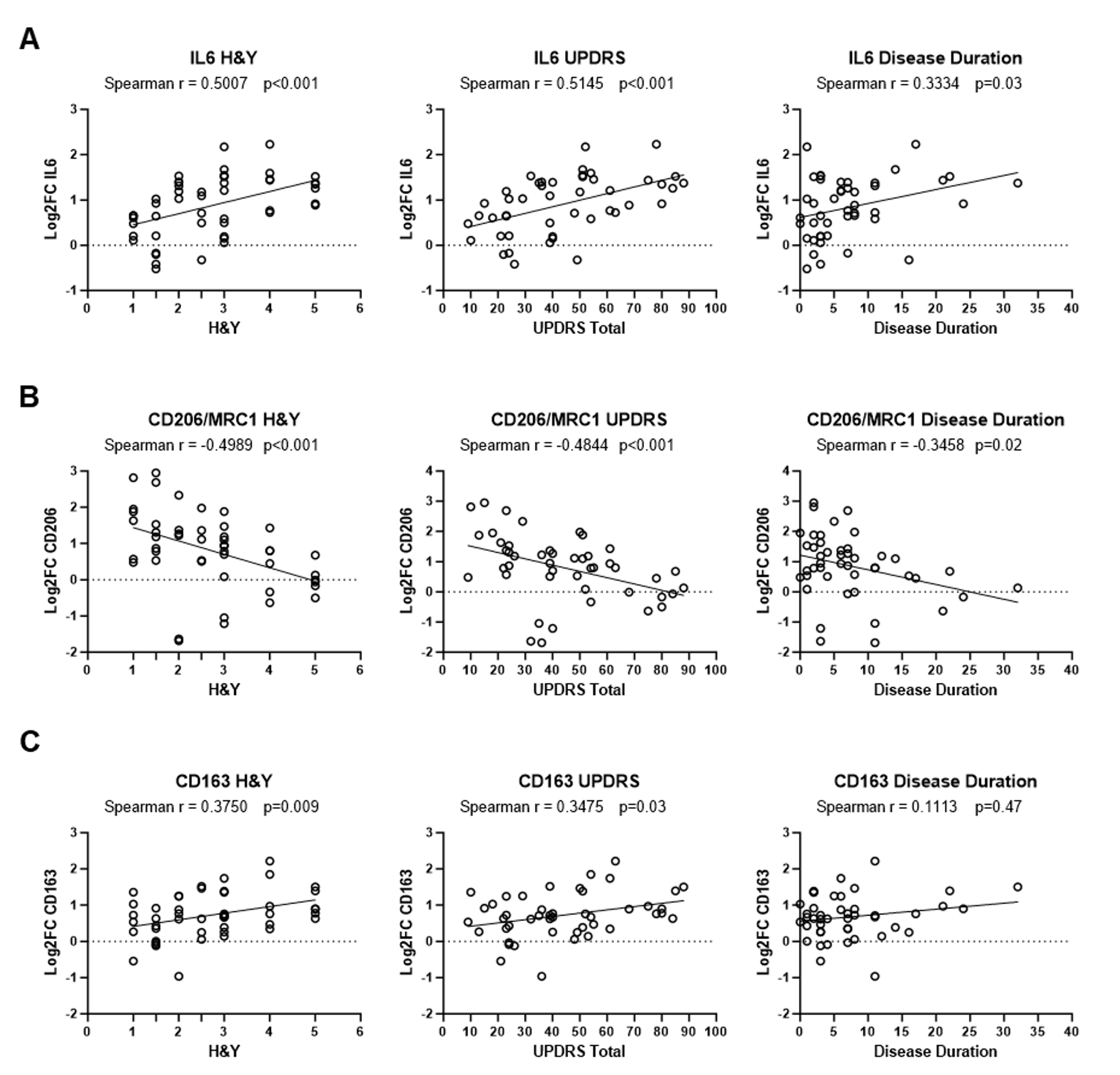

Supplement: Supplementary Figure 1 — PD monocyte inflammatory transcripts correlate with different PD progression parameters. To evaluate correlations with different PD progression parameters, we plotted data and applied simple linear regression modeling to visual trend. Spearman’s correlation coefficient (r) with corresponding p value depicted in each graph with respect to H&Y progression staging, UPDRS scoring, and disease duration via years. Examination of previously significant inflammatory or immunoregulatory transcripts of (A) IL-6, (B) MRC1/CD206, and (C) CD163. [file Image1.tif]

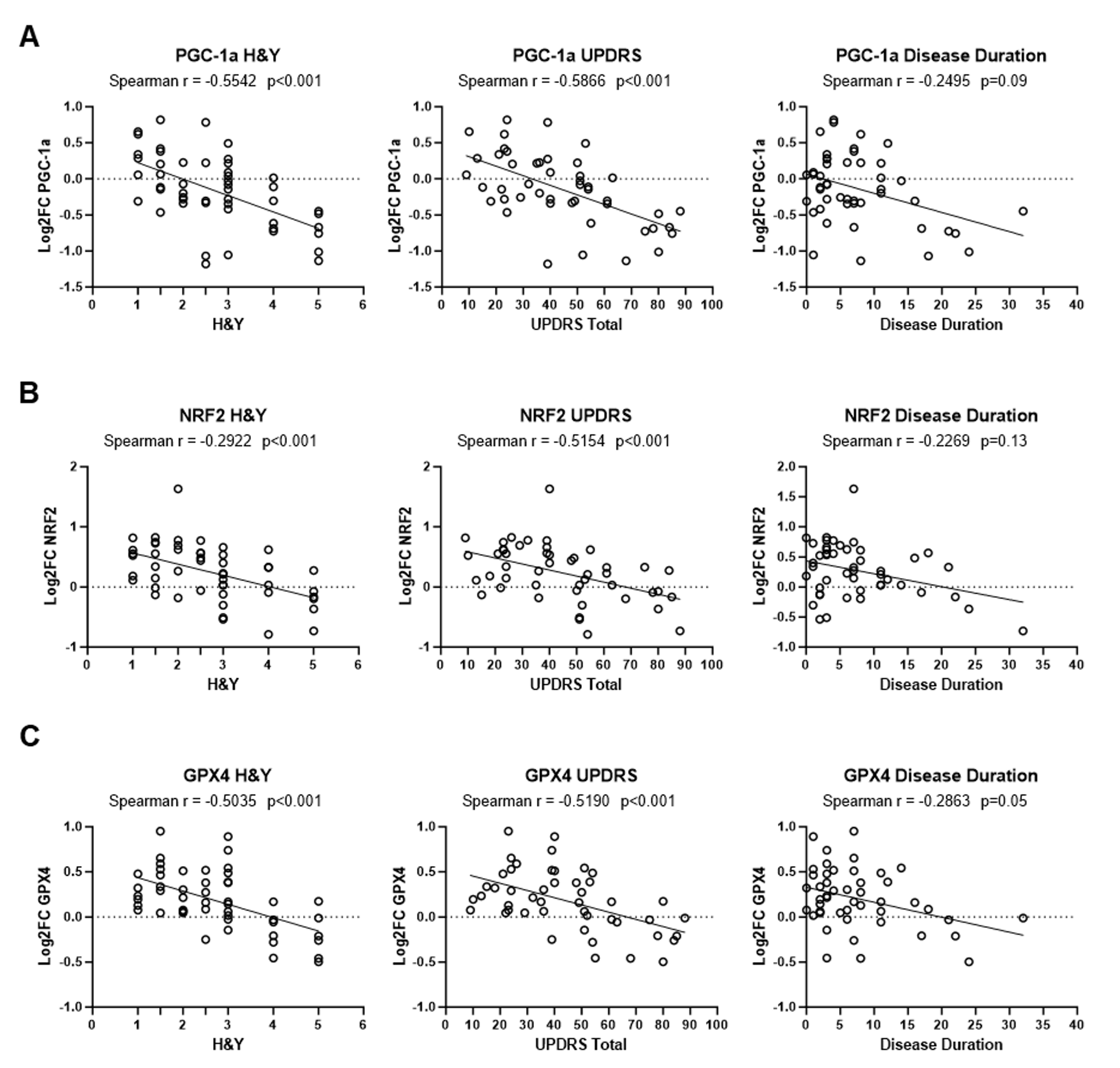

Supplement: Supplementary Figure 2 — PD monocyte oxidative stress transcripts correlate with PD progression parameters. Evaluation of correlations with different PD progression parameters were plotted with applied simple linear regression modeling done to visual trend data. Spearman’s correlation coefficient (r) with corresponding p value depicted in each graph with respect to H&Y progression staging, UPDRS scoring, and disease duration via years. Examination of previously significant inflammatory or immunoregulatory transcripts of (A) PGC-1a, (B) NRF2, and (C) GPX4. [file Image2.tif]
